# Metformin-associated diarrhea in FAERS: a structured pharmacovigilance study with long-term temporal consistency assessment

**DOI:** 10.3389/fphar.2026.1902348

**Published:** 2026-10-09

**Authors:** Shida Chen, Chengan Xu

**Affiliations:** 1 Department of Endocrinology, The Second People’s Hospital of Bengbu City, Bengbu, Anhui, China; 2 Department of Infectious Diseases, Zhejiang Provincial People’s Hospital, Affiliated People’s Hospital, Hangzhou Medical College, Hangzhou, Zhejiang, China

**Keywords:** AERS/FAERS, diarrhea, disproportionality analysis, FAERS, MedDRA, metformin, pharmacovigilance, report-level event-date interval

## Abstract

**Background:**

Metformin-associated diarrhea is well recognized, but spontaneous-report analyses remain sensitive to outcome definition, reporting background, and incomplete event-date data. This study evaluated whether the reporting imbalance persisted across prespecified definitions, comparator sets, and calendar periods.

**Methods:**

U.S. Food and Drug Administration Adverse Event Reporting System (FAERS) reports from the first quarter of 2020 through the first quarter of 2026 were deduplicated and analyzed with metformin coded as a primary or secondary suspect drug. The primary outcome was the Medical Dictionary for Regulatory Activities preferred term DIARRHOEA. Sensitivity analyses used a broad loose-stool phenotype, a clean gastrointestinal event composite, alternative exposure roles, active comparators, co-medication exclusions, and stratification. Exact-date report-level event-date intervals among reports containing the strict-diarrhea preferred term were summarized descriptively. A separately processed dataset covering the first quarter of 2004 through the first quarter of 2026 assessed temporal consistency.

**Results:**

Among 9,112,609 reports, 60,452 involved metformin as a primary or secondary suspect drug and 4,868 included strict diarrhea. The primary reporting odds ratio (ROR) was 2.72 (95% confidence interval, 2.64–2.80). Restriction to non-metformin antidiabetic reports attenuated the ROR to 1.95 (1.89–2.01), showing sensitivity to the reporting background. Only 595 reports (12.2%) yielded a valid report-level event-date interval; the median interval was 14 days (interquartile range, 1–363). The long-term ROR was 2.76 (2.71–2.81), with marked annual variation.

**Conclusion:**

Metformin showed a persistent but context-dependent diarrhea reporting imbalance in FAERS. Active-comparator attenuation limits causal and clinical-risk interpretation. The selected interval subset supports descriptive reporting only and cannot define diarrhea-specific onset or a clinical monitoring window.

## Introduction

Metformin remains a central glucose-lowering therapy for type 2 diabetes because of its established glucose-lowering efficacy, low cost, long clinical experience, and generally favorable clinical profile (American Diabetes Association Professional Practice Committee for Diabetes, 2026). Despite these advantages, gastrointestinal (GI) intolerance remains a common clinical limitation during metformin treatment (Nabrdalik et al., 2022; Bonnet and Scheen, 2017; McCreight et al., 2016). Diarrhea, nausea, vomiting, abdominal discomfort, bloating, and dyspepsia are frequently reported and may contribute to treatment discomfort, dose reduction, poor adherence, or treatment discontinuation (Nabrdalik et al., 2022; Bonnet and Scheen, 2017; Szymczak‐Pajor et al., 2024).

Several mechanisms may contribute to metformin-related GI symptoms. Proposed pathways include high local intestinal drug exposure, altered intestinal glucose utilization, and altered bile acid handling (McCreight et al., 2016; Scarpello et al., 1998). Serotonin-mediated effects may contribute to GI intolerance, and metformin also alters the gut microbiome; the microbiome contribution to intolerance remains uncertain (Cubeddu et al., 2000; Forslund et al., 2015; Wu H. et al., 2017). However, the clinical interpretation of GI symptoms in metformin users is not straightforward. People with diabetes have a high background burden of GI symptoms, and diabetes-related autonomic dysfunction may contribute to diarrhea, constipation, dysmotility, and other bowel symptoms (Vinik et al., 2003; Sommers et al., 2019).

Spontaneous reporting systems, including the U.S. Food and Drug Administration Adverse Event Reporting System (FAERS), provide large-scale post-marketing safety data (U.S. Food and Drug Administration, 2026). These data are useful for signal detection, but they cannot estimate incidence, absolute risk, or causality because they lack a defined exposed denominator and are affected by underreporting, stimulated reporting, duplicate reporting, incomplete clinical information, and reporting bias (U.S. Food and Drug Administration, 2026; Bate and Evans, 2009; Cutroneo et al., 2024). Disproportionality analyses can identify whether specific drug-event pairs are reported more frequently than expected within a spontaneous reporting database, but their results should be interpreted as reporting signals rather than clinical risk estimates (Bate and Evans, 2009; Cutroneo et al., 2024; van Puijenbroek et al., 2002).

A key methodological issue in studying metformin-associated GI adverse-event reporting is outcome definition. Lactic acidosis is a serious metabolic complication associated with metformin in susceptible clinical settings (DeFronzo et al., 2016). Because it is clinically distinct from common GI intolerance, lactic acidosis-related preferred terms were analyzed separately from the clean GI outcomes. Outcomes were coded at the Medical Dictionary for Regulatory Activities (MedDRA) preferred-term (PT) level (Brown et al., 1999).

One recent FAERS study characterized the overall adverse-event profile of metformin (Du et al., 2024). However, diarrhea-specific interpretation requires explicit outcome definitions, transparent disproportionality methods, careful comparator selection, assessment of masking, and cautious causal interpretation (Fusaroli et al., 2024; Gravel et al., 2024; Scosyrev et al., 2025; Bai et al., 2025).

This study addressed those methodological and interpretative needs rather than seeking a previously unknown adverse effect. It used explicit report-level exposure, outcome, deduplication, and date-validity rules. The framework separated lactic acidosis-related events from GI outcomes, tested strict and broad phenotypes across reporting backgrounds, and assessed long-term consistency. Its objectives were to identify when the reporting signal persisted, describe report-level event-date intervals among reports containing the strict-diarrhea PT, and define the limits of spontaneous-report inference.

## Materials and methods

### Data source and study design

This pharmacovigilance study used quarterly FAERS text files from the first quarter of 2020 (2020Q1) through the first quarter of 2026 (2026Q1) as the primary contemporary analysis dataset (U.S. Food and Drug Administration, 2026). FAERS contains spontaneous individual case safety reports submitted by healthcare professionals, consumers, manufacturers, and other reporting sources (U.S. Food and Drug Administration, 2026). The database includes information on patient demographics, reported drugs, adverse event preferred terms, therapy dates, report sources, and clinical outcomes (U.S. Food and Drug Administration, 2026).

This study was designed as a report-level disproportionality analysis (Bate and Evans, 2009; Cutroneo et al., 2024; Van Puijenbroek et al., 2002). Because FAERS is a spontaneous reporting database and does not include a defined exposed population or a complete denominator for metformin use, all estimates were interpreted as reporting signals (U.S. Food and Drug Administration, 2026; Bate and Evans, 2009). They were not interpreted as estimates of incidence, prevalence, absolute risk, relative risk, or causal effects (Bate and Evans, 2009; Cutroneo et al., 2024).

The overall analysis framework is shown in Figure 1. The primary contemporary FAERS dataset and a long-term dataset spanning the legacy Adverse Event Reporting System (AERS) and FAERS were constructed independently. The contemporary dataset was used for the main analysis, whereas the long-term dataset was used to assess temporal consistency of the metformin-diarrhea reporting signal across the broader AERS/FAERS reporting period. Supplementary Table S11 maps each robustness analysis to its scientific purpose and interpretation boundary.

**FIGURE 1:**
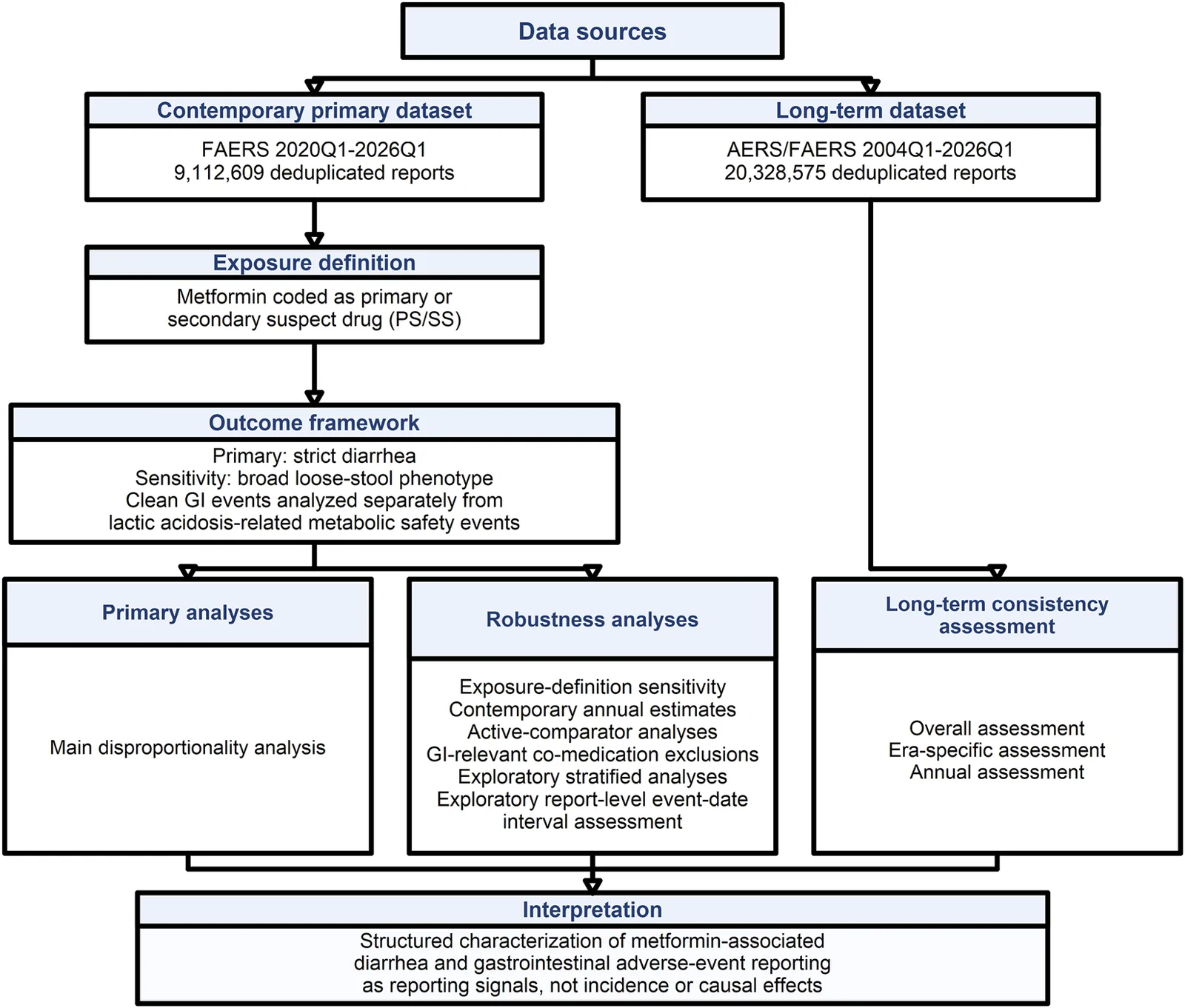
Study design and analysis framework. The contemporary FAERS dataset (2020Q1-2026Q1) supported signal estimation, robustness checks, stratification, and descriptive report-level event-date interval assessment. A separately constructed AERS/FAERS dataset (2004Q1-2026Q1) assessed long-term temporal consistency. The framework distinguishes signal estimation, bias-oriented sensitivity analyses, and descriptive temporal assessment. Abbreviations: AERS, Adverse Event Reporting System; FAERS, U.S. Food and Drug Administration Adverse Event Reporting System; GI, gastrointestinal; PS/SS, primary suspect or secondary suspect; Q, quarter.

### Data import, linkage, and deduplication

Quarterly text files were imported and processed in R. The demographic (DEMO), drug (DRUG), reaction (REAC), outcome (OUTC), report source (RPSR), and therapy (THER) files were linked using the report identifier (PRIMARYID). The case identifier (CASEID) was retained to identify follow-up versions of the same case. After the retained report version was selected, linked tables were filtered to its PRIMARYID and collapsed to one report-level record so that repeated drug or reaction rows did not create duplicate reports.

For the contemporary dataset, DEMO rows missing CASEID or PRIMARYID were excluded before deduplication. Reports were grouped by CASEID and ordered by the latest U.S. Food and Drug Administration receipt date (FDA_DT), with the report date (REPT_DT) used when FDA_DT was missing, followed by the highest case version (CASEVERSION) and then the highest numeric PRIMARYID; the last record in this order was retained. Repeated MedDRA preferred terms within the retained report contributed one report-level event indicator.

Processing checks documented quarterly ingestion, linkage, retained report counts, exposure identification, outcome classification, and analysis-specific exclusions. Supplementary Table S10 provides the complete contemporary and long-term deduplication rules. Supplementary Tables S2–S7, S9 provide the four underlying contingency-table cells for every reported disproportionality estimate, and Supplementary Tables S12, S13 provide the complete operational drug-name and MedDRA preferred-term dictionaries. Core analysis definitions and cross-references are summarized in Supplementary Table S1.

### Metformin exposure definition

Metformin exposure was identified after uppercase standardization by applying a case-insensitive Perl-compatible regular expression to the concatenated drug name (DRUGNAME) and product active ingredient (PROD_AI) fields. The exact metformin tokens were METFORMIN, METFORMIN HCL, METFORMIN HYDROCHLORIDE, GLUCOPHAGE, GLUCOPHAGE XR, FORTAMET, GLUMETZA, and RIOMET. A combination product was classified as metformin-containing only when the same standardized drug row also matched the metformin pattern, commonly through PROD_AI. The complete metformin, comparator-drug, combination-product, and co-medication matching dictionary is provided in Supplementary Table S12.

FAERS drug-role codes were used to define exposure (U.S. Food and Drug Administration, 2026). The primary exposure definition required metformin to be coded as a primary suspect (PS) or secondary suspect (SS) drug. These roles are collectively denoted PS/SS. Sensitivity analyses restricted exposure to primary-suspect reports or expanded it to reports containing metformin under any reported role.

In the PS-present sensitivity analysis, a report qualified when any metformin-matched row was coded PS; other suspect drugs could still be present. A separate report-level sole-suspect sensitivity analysis required at least one metformin-matched PS/SS row and no non-metformin PS/SS row. Concomitant and interacting rows were permitted. Metformin-containing combination rows remained eligible because the drug-role code applies to the individual drug row. A conservative dictionary-restricted analysis additionally excluded flagged fixed-combination rows and reports with unknown non-metformin roles. The corresponding exposure-definition sensitivity results are reported in Supplementary Table S3.

### GI outcome definitions

The primary outcome was strict diarrhea, identified by the MedDRA preferred term DIARRHOEA. The broad loose-stool phenotype contained exactly three preferred terms: DIARRHOEA, FREQUENT BOWEL MOVEMENTS, and FAECES SOFT. Each phenotype was defined at the report level, and repeated reaction rows did not increase the event count. The complete preferred-term dictionary is provided in Supplementary Table S13.

Secondary outcomes included nausea or vomiting, abdominal pain or discomfort, dyspepsia/gas/reflux, constipation, and severe motility-related events. A clean GI event composite included the prespecified GI preferred terms and excluded lactic acidosis-related reactions. These groupings were used to distinguish a common tolerability phenotype from serious metabolic events that require different clinical interpretation.

Lactic acidosis-related reactions included LACTIC ACIDOSIS, HYPERLACTACIDAEMIA, and BLOOD LACTIC ACID INCREASED. They were analyzed as a separate metabolic safety outcome and were excluded from the clean GI event composite. Their disproportionality estimate was not compared directly with symptom-based GI estimates because reporting propensity, clinical severity, and confounding differ substantially.

### Disproportionality analysis

For each exposure-outcome pair, a report-level 2 × 2 contingency table was constructed (Bate and Evans, 2009; van Puijenbroek et al., 2002). The 4 cells were a, exposed-event reports; b, exposed-non-event reports; c, comparator or unexposed-event reports; and d, comparator or unexposed-non-event reports. Supplementary Tables S2–S7, S9 report these cells for the primary, exposure-sensitivity, annual, active-comparator, co-medication exclusion, stratified, and long-term overall/era-specific analyses. The primary comparison was metformin PS/SS reports versus all other reports in the contemporary FAERS dataset. The main measure was the reporting odds ratio (ROR) with a 95% confidence interval (CI). The proportional reporting ratio (PRR), Pearson chi-square statistic, and information component (IC) were supportive measures (Bate and Evans, 2009; van Puijenbroek et al., 2002; Evans et al., 2001; Bate et al., 1998). The IC was calculated as log2{aN/[(a+b)(a+c)]}, where N = a + b + c + d. Its standard error was approximated by the delta method as sqrt[1/a - 1/(a+b) - 1/(a+c) + 1/N]/ln(2). The study-specific Wald lower 95% bound was calculated as the IC minus 1.96 times this standard error. This bound is not the Bayesian lower 95% credibility limit commonly denoted IC025.

ROR positivity was defined *a priori* as a lower 95% confidence limit greater than 1 (Bate and Evans, 2009; van Puijenbroek et al., 2002). The conventional PRR criterion required at least three exposed-event reports, a PRR of at least 2, and a chi-square statistic of at least 4 (Evans et al., 2001). The IC was interpreted as a descriptive supportive measure in spontaneous-reporting signal detection (Bate et al., 1998). An IC Wald lower 95% bound greater than 0 was treated as supportive under a prespecified study-specific rule. For consistency, at least three exposed-event reports were also required for ROR and IC as a study-specific stability rule. These criteria identify reporting disproportionality and do not estimate clinical risk or establish causality (Bate and Evans, 2009; Cutroneo et al., 2024).

### Robustness and sensitivity analyses

Several prespecified analyses addressed different sources of reporting heterogeneity (Fusaroli et al., 2024; Gravel et al., 2024; Scosyrev et al., 2025). Annual estimates assessed whether the primary diarrhea signal was confined to a small number of reporting years. These estimates were descriptive and were not interpreted as annual incidence or a secular trend in clinical risk.

Active-comparator analyses replaced the full FAERS background with reports involving non-metformin antidiabetic drugs. The primary comparator definition accepted eligible comparator drug rows regardless of drug-role code. Secondary pooled and class-specific role-matched sensitivity analyses required at least one eligible comparator-class drug to be coded as PS or SS; all other comparator-report eligibility rules were unchanged. A comparator report was required to contain at least one eligible comparator-class drug and to be free of metformin under every reported role. Reports containing metformin PS/SS and a comparator co-drug remained in the metformin group; reports containing metformin only under another role were excluded from the two-group analysis. Metformin-containing combination products were excluded from comparator groups when DRUGNAME or PROD_AI matched the metformin dictionary. The pooled analysis combined all eligible comparators, and class-specific analyses used glucagon-like peptide-1 (GLP-1) receptor agonists, sodium-glucose cotransporter 2 (SGLT2) inhibitors, dipeptidyl peptidase-4 (DPP-4) inhibitors, alpha-glucosidase inhibitors, insulin, and sulfonylureas. Full class dictionaries and complete 2 × 2 counts are provided in Supplementary Tables S12, S5, respectively.

The six classes were prespecified to provide heterogeneous reporting backgrounds. Class-specific analyses were treated as qualitative sensitivity analyses rather than exchangeable treatment comparisons. Comparator restriction could alter the event frequency in the reference set through class-specific reporting and masking, but it did not adjust for treatment selection or channeling. The primary comparator definition was role-asymmetric because metformin required PS/SS coding while comparator drugs were accepted under any role. The pooled role-matched analysis reduced this coding asymmetry but did not create exchangeable cohorts or adjust for channeling.

Co-medication exclusion analyses removed reports containing selected drugs that could influence GI symptoms or reporting. The prespecified groups comprised GLP-1 receptor agonists, alpha-glucosidase inhibitors, laxatives, magnesium products, antibiotics, opioids, and proton pump inhibitors. Matching used standardized DRUGNAME and PROD_AI fields and the complete token dictionary in Supplementary Table S12. Individual and combined exclusions assessed the sensitivity of the signal to the selected co-reported medication groups. These analyses did not remove unrecorded concomitant therapy or residual confounding. These exclusions were reporting-context sensitivity analyses, not proxy adjustments for indication or channeling. No proxy-adjusted or indication-stratified model was fitted. FAERS indication records contain reporter-supplied treatment indications, but field presence does not establish validated diagnosis, disease severity, treatment line, or clinical comparability. Indication coverage was therefore not treated as evidence that a defensible channeling adjustment could be constructed.

Exploratory analyses were stratified by sex, age group, recorded serious-outcome-code status, reporter-country group, and occurrence-country group. Age values were normalized to years using the reported FAERS age-unit code; values outside 0–120 years or with missing or unsupported units were classified as unknown. Serious-outcome-code strata compared reports with any recorded serious-outcome code with reports with none; absence of a code was not interpreted as confirmed non-seriousness. Their purpose was to describe reporting heterogeneity and identify strata that might warrant future confirmation. All estimable prespecified strata were reported in Supplementary Table S7 with outcome labels and complete 2 × 2 cells so that sample balance could be evaluated. The 37 reports with missing reporter country formed a non-estimable category with no metformin-exposed report and are documented in the table note. These analyses were not designed to identify biologic effect modification, severity attributable to diarrhea, or causal high-risk subgroups.

### Exploratory report-level event-date interval analysis

The exploratory interval analysis was restricted to reports containing the strict-diarrhea PT and an exact day-level report event date (EVENT_DT). Each report also required an exact day-level start date for an eligible metformin row. In the extracted FAERS structure used here, EVENT_DT was recorded at the report level and was not linked to an individual reaction PT. Therefore, the interval cannot be assumed to represent diarrhea-specific onset when a report contains multiple reactions. Metformin rows were identified using the primary exposure dictionary. The earliest valid metformin start date was used when more than one eligible row was present. Partial dates were not imputed.

The report-level event-date interval was calculated as EVENT_DT minus the earliest exact metformin start date. Intervals from 0 through 3,650 days were retained; negative values, impossible dates, and longer intervals were excluded. Summaries included the median, interquartile range (IQR), and proportions at or below 7, 30, 90, and 365 days. An empirical cumulative distribution was displayed through 365 days while retaining each group’s full denominator. This display was not Kaplan-Meier analysis or cumulative incidence because FAERS provides no event-free risk set or censoring process.

Availability of a valid report-level event-date interval was evaluated among all 4,868 eligible metformin PS/SS reports containing the strict-diarrhea PT. Reports contributing a valid interval (n = 595) were compared with the 4,273 noncontributing reports. The comparison included age, sex, recorded serious-outcome-code indicators, report source, report type, reporter occupation and country, reporting year, and numbers of drug, suspect-drug, and reaction records. Continuous and categorical characteristics were summarized using absolute standardized mean differences as the primary descriptive measures of imbalance. Standardized mean differences for record-count variables were calculated after log(1 + x) transformation. Missing categories were retained explicitly. P values were secondary and adjusted across 19 unique tests using the Benjamini-Hochberg false-discovery-rate procedure (Supplementary Table S14). Auxiliary logistic models were not reported because constant and sparse source indicators produced rank-deficient design matrices and unstable standard errors.

Two drug-role sensitivity subsets were examined within the valid-interval cohort. The report-level metformin PS-only subset required every metformin-matched DRUG row to be coded PS but did not exclude non-metformin suspect drugs. The sole-suspect subset excluded reports containing a non-metformin PS/SS row. These overlapping subsets were interpreted descriptively within the full PS/SS valid-interval cohort. The corresponding report-level interval summaries are reported in Supplementary Table S8.

### Long-term temporal consistency assessment

The primary analysis used the contemporary FAERS dataset from 2020Q1 to 2026Q (U.S. Food and Drug Administration, 2026) 1. To evaluate whether the observed metformin-diarrhea reporting signal was limited to the contemporary reporting period or persisted across a broader reporting window, a long-term temporal consistency assessment was conducted using AERS/FAERS data from 2004Q1 to 2026Q1 (U.S. Food and Drug Administration, 2026).

The long-term assessment used a separate harmonized processing workflow. Reports were grouped by standardized CASEID, with PRIMARYID used only when CASEID was missing. The retained record was selected by CASEVERSION and prespecified date and identifier tie-breaks. The same metformin and outcome dictionaries were applied. Strict diarrhea was assessed overall, across three reporting eras, and annually from 2004 through 2026Q1. For Figure 2, centered 3-year pooled RORs were calculated by summing the four 2 x 2 cells across complete calendar years and recalculating the ROR and Wald 95% CI. Windows ran from 2004–2006 through 2023–2025 and were assigned to their middle year. The incomplete 2026Q1 period was excluded from this descriptive smoother. Complete annual and centered 3-year pooled estimates are reported in Supplementary Table S9.

**FIGURE 2:**
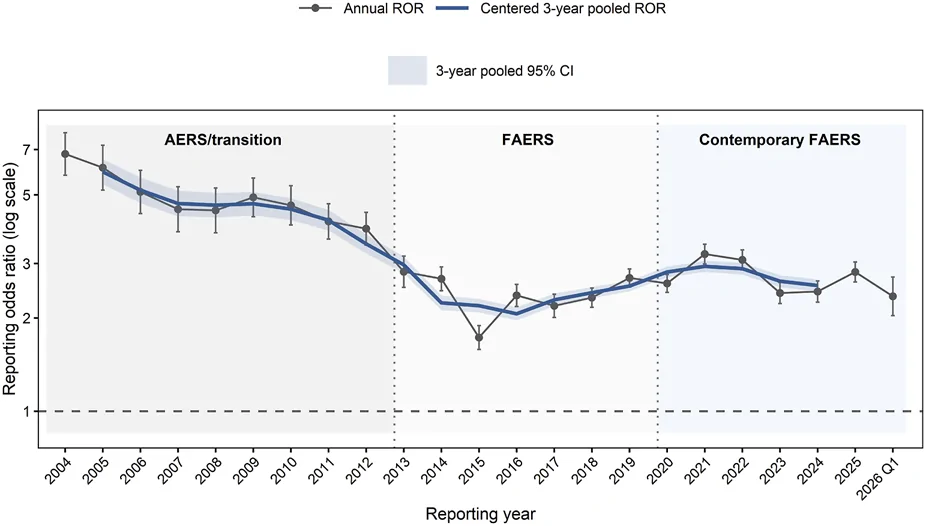
Long-term temporal consistency assessment of metformin-associated strict-diarrhea reporting in AERS/FAERS. Gray points and 95% CIs show annual RORs from 2004 through 2026Q1. The blue line and band show centered 3-year pooled RORs and 95% CIs from complete calendar-year windows ending in 2025; 2026Q1 is excluded from the pooled line. Era boundaries are marked. The smoother is descriptive and does not remove reporting-system heterogeneity or establish external replication. Abbreviations: AERS, Adverse Event Reporting System; CI, confidence interval; FAERS, U.S. Food and Drug Administration Adverse Event Reporting System; PS/SS, primary suspect or secondary suspect; Q, quarter; ROR, reporting odds ratio.

The long-term analyses were conducted as temporal consistency assessments and did not replace the contemporary primary analysis. Because spontaneous-reporting data are influenced by reporting behavior, database composition, and the available comparator background, era-specific and annual estimates were interpreted as reporting patterns rather than changes in incidence or clinical risk (U.S. Food and Drug Administration, 2026; Bate and Evans, 2009; Cutroneo et al., 2024).

The contemporary primary analysis dataset and the long-term dataset were constructed separately and used different archived deduplication implementations. In the overlapping 2020Q1-2026Q1 period, the contemporary workflow retained 9,112,609 reports, whereas the harmonized long-term workflow retained 9,112,220 reports, a difference of 389 reports (0.004%). The difference reflects missing-identifier handling and tie-break priority, not different outcome definitions; both segments contained 4,868 metformin PS/SS diarrhea reports and yielded virtually identical RORs.

### Ethics statement

This study used publicly available, deidentified spontaneous adverse‐event reporting data. No identifiable human participant data were accessed. The Medical Ethics Committee of The Second People's Hospital of Bengbu City determined that the study met the criteria for exemption from ethics review (ethics review opinion letter, No. 8 [2026]).

### Statistical software

All analyses were performed using R version 4.6.1 (R Core Team, 2026). Data processing used data.table version 1.18.4, and figures were generated using ggplot2 version 4.0.3 (Barrett et al., 2026; Wickham, 2016).

## Results

### Analysis population

The primary contemporary analysis included 9,112,609 deduplicated FAERS reports from 2020Q1 to 2026Q1. Metformin was coded as a PS/SS drug in 60,452 reports. Table 1 shows that 4,868 of these reports included the MedDRA preferred term DIARRHOEA.

**TABLE 1:** Selected contemporary disproportionality findings for metformin PS/SS reports in FAERS, 2020Q1-2026Q1.

Outcome	Metformin PS/SS event reports	ROR (95% CI)	PRR	IC lower (Wald 95%)	Interpretation
Strict diarrhea	4,868	2.72 (2.64–2.80)	2.58	1.31	Positive primary signal
Broad loose-stool phenotype	5,123	2.76 (2.68–2.84)	2.61	1.33	Positive sensitivity signal
Clean GI event composite	11,010	2.16 (2.11–2.20)	1.95	0.93	Positive ROR/IC; PRR below 2
Severe motility-related event	136	0.89 (0.76–1.06)	0.89	−0.40	No positive signal
Lactic acidosis-related metabolic safety event	11,251	280.11 (271.65–288.83)	228.16	6.50	Separate metabolic safety signal

FAERS, U.S. Food and Drug Administration Adverse Event Reporting System; PS/SS, primary suspect or secondary suspect; Q, quarter; GI, gastrointestinal; ROR, reporting odds ratio; CI, confidence interval; PRR, proportional reporting ratio; IC, information component. The IC lower (Wald 95%) column gives the approximate delta‐method Wald lower 95% confidence bound for the information component. Lactic acidosis-related events were analyzed as metabolic safety outcomes and were not included in the clean GI event composite.

### Primary disproportionality findings

Metformin PS/SS reports showed disproportionate reporting of strict diarrhea. Table 1 and Figure 3 show that the ROR was 2.72 (95% CI, 2.64–2.80), the PRR was 2.58, and the approximate IC Wald lower 95% confidence bound was 1.31. Complete contingency-table cells for all primary phenotypes are provided in Supplementary Table S2.

**FIGURE 3:**
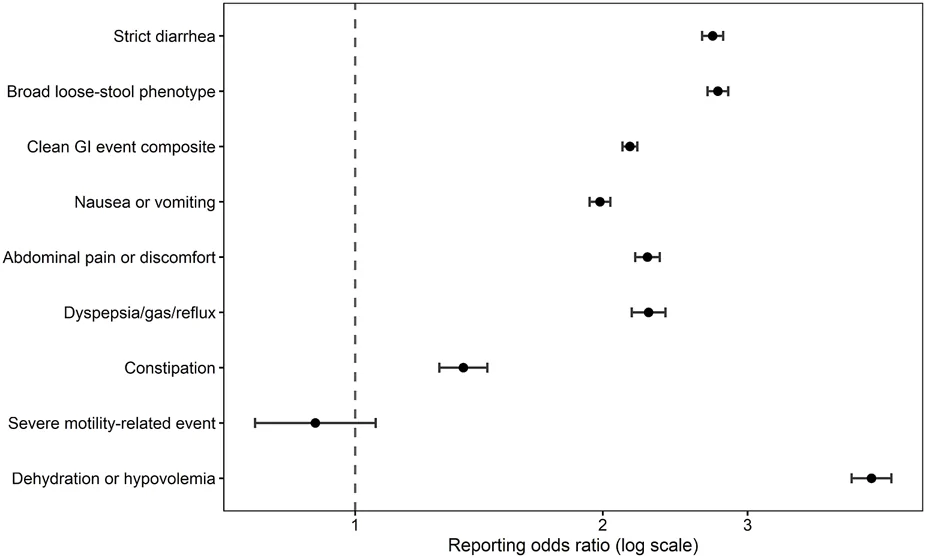
Main disproportionality signals for metformin-associated GI and selected safety outcomes in contemporary FAERS. Points show reporting odds ratios (RORs), and horizontal lines show 95% confidence intervals; the vertical line indicates ROR = 1. Strict diarrhea and the broad loose-stool phenotype showed positive reporting signals, whereas the severe motility-related event did not meet the prespecified criterion. The lactic acidosis-related ROR (280.11) is reported in Table 1 but is not plotted because its scale would compress the GI estimates; its magnitude must not be interpreted as event frequency or ranked directly against symptom outcomes. Abbreviations: FAERS, U.S. Food and Drug Administration Adverse Event Reporting System; GI, gastrointestinal; ROR, reporting odds ratio.

The finding was consistent when the broader loose-stool phenotype was used, with an ROR of 2.76. The clean GI event composite had a positive ROR signal and positive IC signal, but its PRR was 1.95 and therefore fell just below the prespecified PRR threshold of 2. This metric-level discrepancy is expected because ROR, PRR, and IC use different reference quantities and decision thresholds; the composite is therefore interpreted as a positive but metric-dependent reporting pattern rather than a criterion-independent signal. Secondary GI outcomes also showed positive ROR signals, whereas severe motility-related events did not.

Lactic acidosis-related events showed a strong separate metabolic safety signal in Table 1. These events were analyzed separately and were not included in the clean GI event composite.

### Robustness analyses in contemporary FAERS

Key robustness and exploratory findings are summarized in Table 2; Figure 4 separates active-comparator and co-medication analyses. Against any non-metformin antidiabetic report, the strict-diarrhea ROR was 1.95 (95% CI, 1.89–2.01). Class-specific RORs ranged from 1.28 for sulfonylureas to 3.18 for insulin, showing that comparator choice influenced signal magnitude. Complete class-specific counts are provided in Supplementary Table S5. Restriction to reports with any PS-coded metformin row yielded an ROR of 2.50 (95% CI, 2.40–2.59; Supplementary Table S3). This role-based sensitivity analysis did not require metformin to be the only suspect drug.

**TABLE 2:** Key robustness and exploratory analyses for metformin-associated diarrhea in contemporary FAERS, 2020Q1-2026Q1.

Analysis	Result	Interpretation
Active comparator: Any-role and role-matched non-metformin antidiabetic-drug reports	Any role: ROR, 1.95 (95% CI, 1.89–2.01); PS/SS role-matched: ROR, 2.11 (95% CI, 2.04–2.18)	Role matching reduces role-code asymmetry but does not establish exchangeability, channeling adjustment, or comparative safety
Combined GI-relevant co-medication exclusion	ROR, 2.75 (95% CI, 2.65–2.85)	Signal persisted after excluding reports with selected GI-relevant co-reported medications
Exploratory report-level event-date interval	595 valid intervals; median, 14 days; IQR, 1–363; 75.1% were 365 days or shorter; report-level metformin PS-only subset, n = 390, median, 9 days (IQR, 0–304); sole-suspect subset, n = 400, median, 11 days (IQR, 0–153); coverage, 12.2%	Selected reports containing the strict-diarrhea preferred term; the event date is report-level and not reaction-specific; not diarrhea-specific onset, cumulative incidence, or a clinical monitoring window
Stratified analyses	Positive signals across sex, age group, recorded serious-outcome-code status, reporter-country group, and occurrence-country group	Exploratory reporting heterogeneity only; not causal effect modification, event-specific severity, or a high-risk subgroup analysis

FAERS, U.S. Food and Drug Administration Adverse Event Reporting System; Q, quarter; GI, gastrointestinal; ROR, reporting odds ratio; CI, confidence interval; IQR, interquartile range; n, number of reports; PS, primary suspect; PS/SS, primary suspect or secondary suspect. Active-comparator and co-medication exclusion analyses used the strict diarrhea outcome. Stratified estimates describe reporting heterogeneity and should not be interpreted as causal subgroup effects. Report-level event-date interval estimates were based on the 12.2% of eligible metformin PS/SS reports containing the strict-diarrhea preferred term that yielded valid intervals. The pooled role-matched comparator sensitivity is reported in Supplementary Table S5.

**FIGURE 4:**
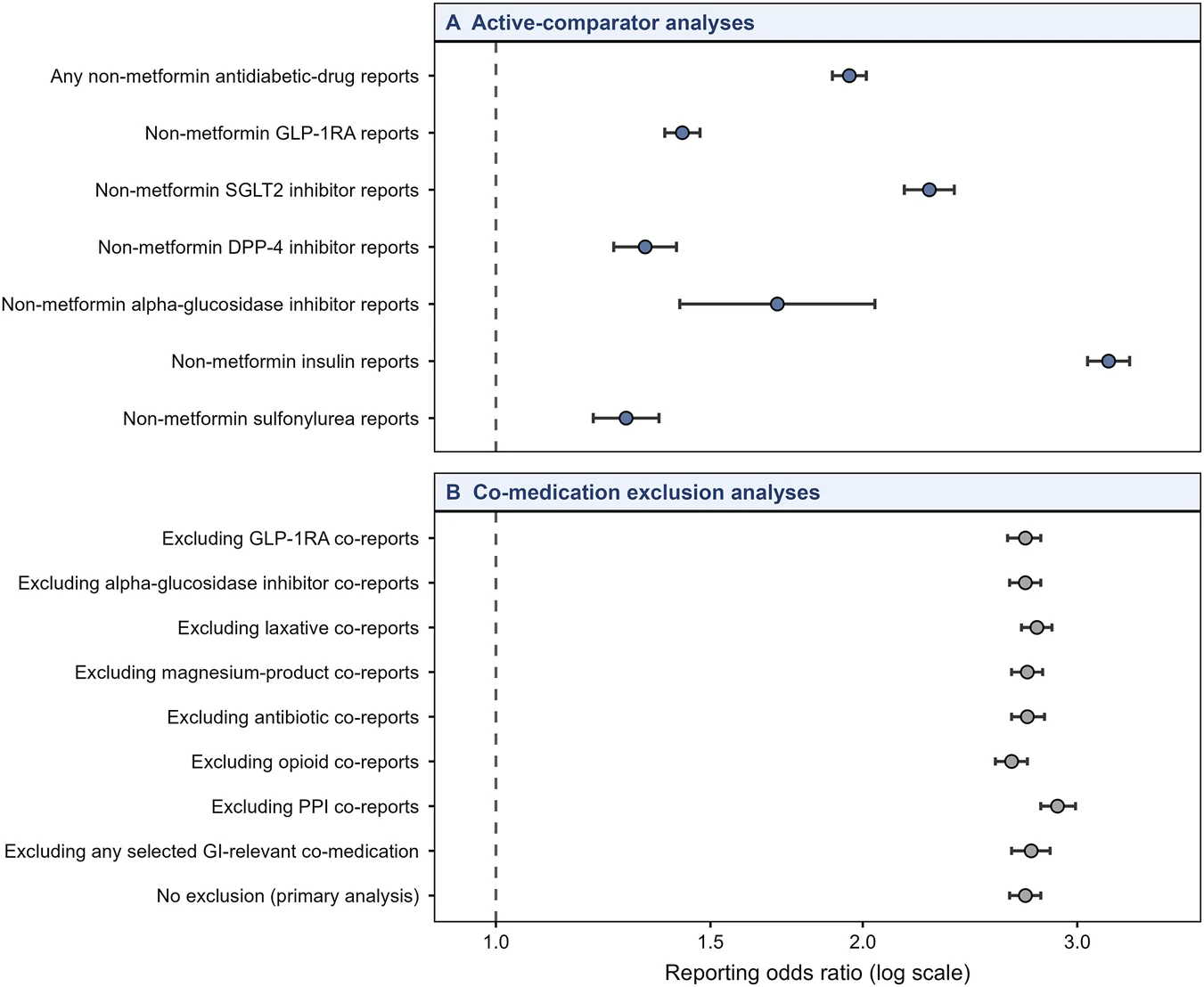
Robustness analyses for metformin-associated strict diarrhea in contemporary FAERS. Panel A shows active-comparator estimates, and panel B shows co-medication exclusion estimates. Points are RORs and horizontal lines are 95% CIs; the vertical dashed line marks ROR = 1. Attenuation and between-class variation show that signal magnitude depends on the reporting background and is not a comparison of clinical diarrhea risk. Abbreviations: CI, confidence interval; DPP-4, dipeptidyl peptidase-4; FAERS, U.S. Food and Drug Administration Adverse Event Reporting System; GI, gastrointestinal; GLP-1RA, glucagon-like peptide-1 receptor agonist; PPI, proton pump inhibitor; ROR, reporting odds ratio; SGLT2, sodium-glucose cotransporter 2. The pooled role-matched comparator sensitivity analysis is reported in Supplementary Table S5 and is not plotted.

When comparator eligibility was restricted to PS/SS-coded non-metformin antidiabetic drugs, the pooled role-matched ROR was 2.11 (95% CI, 2.04–2.18; c = 17,125, d = 412,432; Supplementary Table S5). This analysis assessed sensitivity to comparator role coding but did not establish exchangeability or comparative safety.

Under the report-level sole-suspect definition, the strict-diarrhea ROR was 2.39 (95% CI, 2.28–2.51; a = 1,817, b = 23,463, c = 285,389, d = 8,801,940). The conservative dictionary-restricted definition produced an ROR of 2.59 (2.46–2.73). Both analyses remained positive but did not establish causal attribution (Supplementary Table S3).

After the combined co-medication exclusion, the ROR was 2.75 (95% CI, 2.65–2.85), and individual exclusions produced similar estimates (Supplementary Table S6). Exploratory estimates were also positive across strata defined by sex, age group, recorded serious-outcome-code status, reporter country, and occurrence country. These differences were treated as reporting heterogeneity rather than causal susceptibility or event-specific severity. Complete stratified results are provided in Supplementary Table S7 and visualized in Supplementary Figure S1. Long-term annual patterns for the prespecified outcomes are shown in Supplementary Figure S2.

### Exploratory report-level event-date interval analysis

A valid report-level event-date interval was available for 595 of 4,868 metformin PS/SS reports containing the strict-diarrhea PT (12.2%). The median interval was 14 days (IQR, 1–363), and 447 of 595 intervals (75.1%) were 365 days or shorter. In the report-level metformin PS-only subset (n = 390), the median interval was 9 days (IQR, 0–304), and 296 of 390 intervals (75.9%) were 365 days or shorter. The complementary 205 metformin PS/SS interval contributors that did not meet the report-level PS-only definition had a median interval of 20 days (IQR, 1–377), and 151 of 205 intervals (73.7%) were 365 days or shorter. In the sole-suspect subset (n = 400), the median interval was 11 days (IQR, 0–153), and 312 of 400 intervals (78.0%) were 365 days or shorter. Supplementary Table S8 and Supplementary Figure S3 provide the full distributions. Because EVENT_DT is report-level and not reaction-specific, these intervals cannot establish diarrhea-specific onset or a clinical monitoring window.

Measured characteristics differed substantially between reports contributing and not contributing a valid report-level event-date interval (Supplementary Table S14). Contributors had fewer unique reaction preferred terms (median, 4 [IQR, 2–6] vs. 6 [3–14]; absolute standardized mean difference, 0.634) and fewer suspect-drug records (1 [1–2] vs. 3 [1–7]; 0.632). Age was unavailable less often (11.4% vs. 31.3%; 0.500), and health professionals were more frequent reporters (49.9% vs. 27.8%; 0.466). A recorded serious-outcome code was more common (85.9% vs. 75.8%; 0.259), and the RPSR source field was more often populated (no source recorded, 87.1% vs. 94.2%; 0.248). These imbalances indicate measured selection into the valid-interval subset.

### Long-term and annual temporal consistency assessments

The long-term dataset was constructed separately from the contemporary dataset. It included 20,328,575 reports from 2004Q1 through 2026Q1. Metformin was coded as PS/SS in 138,893 reports, including 11,016 strict-diarrhea reports. The overall ROR was 2.76 (95% CI, 2.71–2.81), the PRR was 2.62 (2.57–2.67), and the approximate IC Wald lower 95% confidence bound was 1.35 (Table 3). The overlapping 2020Q1-2026Q1 segment differed from the contemporary workflow by 389 retained reports but contained the same 4,868 metformin-diarrhea reports.

**TABLE 3:** Long-term AERS/FAERS temporal consistency assessment of the metformin-diarrhea reporting signal.

Analysis	Period	Reports, n	Metformin PS/SS, n	Metformin PS/SS strict diarrhea, n	ROR (95% CI)	PRR (95% CI)	IC lower (Wald 95%)
Overall	2004Q1-2026Q1	20,328,575	138,893	11,016	2.76 (2.71–2.81)	2.62 (2.57–2.67)	1.35
Era-specific	AERS 2004Q1-2012Q3	3,008,852	15,155	1,744	4.78 (4.55–5.03)	4.35 (4.16–4.55)	2.03
Era-specific	FAERS 2012Q4-2019Q4	8,207,503	63,293	4,404	2.36 (2.29–2.44)	2.27 (2.21–2.34)	1.13
Era-specific	FAERS 2020Q1-2026Q1, overlapping long-term dataset segment	9,112,220	60,445	4,868	2.72 (2.64–2.80)	2.58 (2.51–2.65)	1.31

AERS, Adverse Event Reporting System; FAERS, U.S. Food and Drug Administration Adverse Event Reporting System; Q, quarter; n, number of reports; PS/SS, primary suspect or secondary suspect; ROR, reporting odds ratio; CI, confidence interval; PRR, proportional reporting ratio; IC, information component. The IC lower (Wald 95%) column gives the approximate delta‐method Wald lower 95% confidence bound for the information component. Metformin PS/SS reports were those in which metformin was coded as a primary or secondary suspect drug. The 2020Q1-2026Q1 row represents the overlapping contemporary segment of the separately constructed long-term dataset. Cross-era differences are interpreted as reporting-system heterogeneity, not as changes in incidence.

The reporting signal was positive in each predefined reporting era. The ROR was 4.78 (95% CI, 4.55–5.03) in AERS 2004Q1-2012Q3. It was 2.36 (2.29–2.44) in FAERS 2012Q4-2019Q4 and 2.72 (2.64–2.80) in FAERS 2020Q1-2026Q1 (Table 3).

Annual strict-diarrhea RORs ranged from 1.73 (95% CI, 1.58–1.89) in 2015 to 6.77 (5.78–7.93) in 2004, and every lower confidence limit exceeded 1. The centered 3-year pooled ROR declined from 5.93 (5.40–6.51) for 2004–2006 to 2.06 (1.97–2.17) for 2015–2017, then reached 2.55 (2.44–2.66) for 2023–2025. Figure 2 and Supplementary Table S9 retain the raw annual estimates and show this descriptive count-pooled smoother. The incomplete 2026Q1 period appears only as an annual point.

## Discussion

### Principal findings and incremental contribution

The study’s incremental value is the coordination of three contributions rather than discovery of an unknown adverse effect. Methodologically, it applies a layered antidiabetic-drug pharmacovigilance framework that combines phenotype definition, active comparators, co-medication exclusions, exposure sensitivity analyses, and transparent 2 × 2 data. As a data contribution, it evaluates temporal persistence across a separately constructed 2004Q1-2026Q1 AERS/FAERS record. As an application and interpretation contribution, it operationally separates common GI tolerability outcomes from lactic acidosis-related metabolic safety events and reports the completeness and interpretation boundaries of report-level event-date intervals. These elements define how a known signal can be characterized reproducibly without converting spontaneous reports into incidence or causal evidence.

Within the contemporary dataset, diarrhea was reported disproportionately for metformin PS/SS reports. The estimate was stable under a broader loose-stool definition and co-medication exclusions. It was attenuated, but remained positive, when the comparator was restricted to other antidiabetic drugs. The long-term analysis showed that the signal was present throughout AERS/FAERS, although its magnitude varied over time.

### Comparison with prior evidence

The findings are consistent with clinical evidence that diarrhea and other GI symptoms are frequent during metformin therapy (Cheng et al., 2024; Nabrdalik et al., 2024). A recent FAERS study evaluated the wider adverse-event spectrum of metformin (Du et al., 2024). The present study narrows the question to diarrhea and adds explicit phenotype separation, diabetes-drug comparator analyses, co-medication exclusions, valid report-level event-date interval coverage, and a separately constructed long-term dataset. Recent methodological guidance on transparent reporting, comparator selection, masking, and causal limits supports interpreting these additions as design-based signal characterization rather than risk estimation (Fusaroli et al., 2024; Gravel et al., 2024; Scosyrev et al., 2025; Bai et al., 2025).

The attenuation from the whole-database ROR of 2.72 to the pooled antidiabetic-comparator ROR of 1.95 is compatible with partial control of diabetes-related reporting context, but it is not evidence that clinical diarrhea risk decreased. Class-specific estimates were lowest against sulfonylurea (1.28), DPP-4 inhibitor (1.33), and GLP-1 receptor agonist (1.42) reports and highest against insulin reports (3.18), with intermediate estimates for alpha-glucosidase inhibitors (1.70) and SGLT2 inhibitors (2.27). These differences describe distinct reporting backgrounds rather than a ranking of the comparator drugs’ clinical GI safety. Restricted reference sets can reduce some sources of bias while introducing class-specific reporting and masking effects, so the full comparator panel is more informative than any single estimate (Gravel et al., 2024; Scosyrev et al., 2025; Bai et al., 2025).

Comparator-class GI profiles provide a qualitative expectation for bias. GI adverse events are prominent with GLP-1 receptor agonists and alpha-glucosidase inhibitors, whereas DPP-4 inhibitors generally have a more neutral GI profile (Sun et al., 2015; Wu S. et al., 2017; Maruthur et al., 2016; van de Laar et al., 2005).

A GI-active comparator may increase diarrhea reporting in the reference set and thereby attenuate or mask the metformin ROR. Comparisons with insulin or sulfonylureas may also be affected by unmeasured treatment-stage differences. These possibilities were treated as qualitative bias hypotheses rather than measured effects. Confounding by indication and channeling remain because treatment line, diabetes duration, glycemic control, renal function, comorbidity, and polypharmacy were unavailable. The comparator analyses therefore do not create exchangeable cohorts.

The primary ROR of 2.72 denotes a reporting imbalance relative to the selected background. It is not a 2.72-fold clinical risk or a severity category. Raw RORs from different databases, periods, or outcome definitions are not calibrated for cross-study ranking. The within-study attenuation to 1.95 is the relevant evidence that the signal magnitude depends on the reporting background.

### Mechanistic context for gastrointestinal intolerance

Metformin reaches high concentrations in the intestinal lumen and wall, and several pathways may contribute to gastrointestinal intolerance. Proposed mechanisms include altered bile acid handling, increased intestinal serotonin signaling, and metformin-associated changes in the gut microbiota (McCreight et al., 2016; Scarpello et al., 1998; Cubeddu et al., 2000; Forslund et al., 2015; Wu H. et al., 2017; Cheng et al., 2024). These findings provide biological context, but the contribution of microbiome changes to diarrhea remains uncertain. Recent work identified intestinal epithelial mitochondrial complex I as an important site of metformin action; however, that study addressed glycemic control rather than metformin-associated diarrhea (Sebo et al., 2026). These mechanisms provide biological context but do not establish the cause of an individual FAERS report.

### Outcome-specific clinical interpretation

The clean GI event composite excluded lactic acidosis-related reactions because the two outcomes represent different clinical problems. Diarrhea usually reflects tolerability, whereas metformin-associated lactic acidosis is a distinct emergency associated with renal impairment, acute illness, tissue hypoxia, and drug accumulation (See, 2024; Kim et al., 2024). A large ROR for lactic acidosis therefore does not imply that it is more common than diarrhea, and the two RORs should not be ranked as clinical risks.

The severe motility-related event did not meet the prespecified positive-signal criterion, but this finding does not exclude severe diarrhea. In the extracted FAERS structure used here, seriousness and outcome fields were linked to the report rather than to individual reaction PTs. Consequently, they cannot establish that diarrhea caused hospitalization or another serious outcome in a multireaction report (Cutroneo et al., 2024). Accordingly, this study does not identify diarrhea-specific severity or high-risk subgroups.

### Temporal heterogeneity and long-term consistency

Higher RORs in the early AERS years should not be interpreted as higher clinical risk. Spontaneous-reporting estimates can vary with reporting behavior, database composition, calendar time, and the available comparator background. Potential contributors include changes in AERS/FAERS file structures, submission volume, reporting channels, and MedDRA coding or follow-up practices. Changes in clinical recognition, metformin utilization, and the expanding antidiabetic treatment landscape may also alter the reporting background (U.S. Food and Drug Administration, 2026; Bate and Evans, 2009; Cutroneo et al., 2024; Gravel et al., 2024; Scosyrev et al., 2025; Bai et al., 2025). These factors can affect both the numerator and the reporting background, but their individual contributions cannot be quantified because FAERS lacks utilization denominators.

These drivers were not empirically decomposed. They are presented as plausible sources of reporting-system heterogeneity, not as mechanisms established by this study.

The value of the long-term analysis is temporal persistence within the same reporting system, not quantitative equivalence or external replication. The centered 3-year line reduces visual noise but cannot correct structural changes in AERS/FAERS, treatment utilization, or comparator composition. Annual and rolling variation is therefore interpreted as reporting-system heterogeneity rather than changing clinical incidence.

### Active-comparator and stratified analyses

Taken together, the pooled and class-specific analyses show that no single reference set is definitive. Restriction to antidiabetic reports may improve comparability of indication and healthcare context, but class-specific reporting patterns and masking may remain (Gravel et al., 2024; Scosyrev et al., 2025; Bai et al., 2025). Reporting the full comparator panel therefore provides a more transparent assessment of reference-group sensitivity than emphasizing any single estimate.

Expected comparator-related bias was assessed qualitatively from class-level gastrointestinal profiles. GLP-1 receptor agonists and alpha-glucosidase inhibitors commonly generate gastrointestinal adverse-event reports, whereas DPP-4 inhibitors generally have a more neutral gastrointestinal profile. For SGLT2 inhibitors, sulfonylureas, and insulin, possible effects of treatment selection, concomitant therapy, and report composition were treated as unquantified hypotheses rather than established directions of bias. The cited literature supports the class-level gastrointestinal profiles but does not identify the magnitude or direction of reporting bias (Sun et al., 2015; Wu S. et al., 2017; Maruthur et al., 2016; van de Laar et al., 2005).

This direction-of-bias assessment is qualitative. The observed class-specific RORs do not estimate comparator-induced bias. Without treatment denominators and measured variables for treatment line, comorbidity, concomitant therapy, and report selection, neither the magnitude of class-specific bias nor a bias-corrected clinical effect is identifiable from FAERS.

The pooled role-matched ROR was 2.11 (95% CI, 2.04–2.18), compared with 1.95 under the any-role comparator definition. This sensitivity analysis reduced one coding asymmetry but did not resolve confounding by indication, channeling, treatment-stage differences, or selective reporting.

The stratified analyses served a descriptive purpose. Positive estimates across strata defined by demographic variables, recorded serious-outcome-code status, and geography suggest that the overall signal was not confined to one recorded subgroup. However, missing covariates, small cells in some strata, multiple comparisons, and the absence of patient-level denominators prevent ranking strata as clinically high risk or making causal claims about susceptibility. Confirmation would require linked clinical data with dose, formulation, renal function, comorbidity, and follow-up.

### Clinical and pharmacovigilance implications

Within the selected valid-interval subset, 59.0% of report-level event-date intervals were 30 days or shorter, and 75.1% were 365 days or shorter. These values describe reports containing the strict-diarrhea PT, not confirmed diarrhea-specific onset. EVENT_DT is recorded at the report level and may refer to another reaction in a multireaction report. The subset represented 12.2% of eligible reports and differed from noncontributing reports across several measured characteristics. These limitations prevent the interval distribution from defining a high-risk period or clinical monitoring schedule. Future pharmacovigilance data should link reaction-specific onset dates to drug initiation, dose changes, and symptom resolution.

Clinicians should distinguish expected early gastrointestinal intolerance from symptoms that warrant broader evaluation. During metformin initiation or dose escalation, gradual titration, administration with meals, and consideration of an extended-release formulation may improve tolerability (American Diabetes Association Professional Practice Committee for Diabetes, 2026; Bonnet and Scheen, 2017). Persistent or severe diarrhea and dehydration warrant clinical assessment. Acute kidney injury, hypoxia, or systemic illness also warrant evaluation for conditions beyond uncomplicated gastrointestinal intolerance, including suspected lactic acidosis (DeFronzo et al., 2016; See, 2024; Kim et al., 2024). These management statements derive from prior clinical evidence and guideline recommendations, not from the FAERS report-level event-date interval analysis.

### Strengths and limitations

This study has several strengths. It used a large contemporary FAERS dataset; explicit report-level deduplication; separate strict, broad, and metabolic phenotypes; fully specified drug and reaction dictionaries; complete 2 × 2 counts; operationally defined active comparators; multiple co-medication and exposure sensitivity analyses; and a separately constructed long-term dataset. It also reports valid-interval coverage and the full descriptive report-level event-date interval distribution rather than presenting an interval estimate without its denominator.

Several limitations are intrinsic to the data source. Spontaneous reports are subject to underreporting, stimulated and differential reporting, incomplete or duplicate submissions, and coding errors (Bate and Evans, 2009; Cutroneo et al., 2024; Fusaroli et al., 2024). Because FAERS lacks exposure denominators, disproportionality measures estimate reporting imbalance rather than incidence, absolute risk, or causality (Bate and Evans, 2009; Cutroneo et al., 2024; Scosyrev et al., 2025).

Study-specific limitations add to these general constraints. Notoriety bias remains possible because metformin-associated diarrhea is well recognized, which may increase recognition or reporting of the drug-event pair (Bonnet and Scheen, 2017; Cutroneo et al., 2024). Active comparators and co-medication exclusions can reduce selected differences in reporting context but cannot eliminate stimulated reporting, masking, or residual confounding (Gravel et al., 2024; Scosyrev et al., 2025; Bai et al., 2025). FAERS also lacked or inconsistently recorded important clinical variables, including glycemic control, diabetes duration, metformin dose and formulation, titration, adherence, diet, preexisting gastrointestinal disease, renal function, infection, and alternative causes of diarrhea.

Confounding by indication and channeling may arise from treatment line, disease duration, glycemic control, renal function, comorbidity, and polypharmacy. These determinants were unavailable or not reliably measured in the retained data. The presence or coverage of reporter-entered indication records was not treated as evidence that channeling adjustment was feasible. The co-medication exclusions should not be interpreted as proxy adjustment. Active comparators reduce selected reporting-context differences but do not form exchangeable cohorts or support causal adjustment. These statements describe unresolved bias mechanisms rather than empirically established class effects.

Only 12.2% of eligible reports yielded a valid report-level event-date interval; therefore, the interval distribution may be affected by selection bias and should not be generalized to noncontributing reports. In the extracted FAERS structure, case-level seriousness fields were not linked to individual reaction PTs; combined with spontaneous-reporting limitations, this prevents attributing a serious outcome to diarrhea in multireaction reports (Cutroneo et al., 2024; Scosyrev et al., 2025). Accordingly, this study does not estimate diarrhea-specific severity, prognosis, or causal high-risk subgroups. Because EVENT_DT is report-level rather than reaction-specific, the interval cannot establish diarrhea-specific onset in multireaction reports.

The report-level comparison identified substantial measured selection into the valid-interval subset, but it could not evaluate unrecorded determinants of date availability. The sole-suspect restriction reduced ambiguity from co-suspect drug rows but did not address underreporting, stimulated reporting, masking, confounding, or event attribution. Dose and formulation were not analyzed because free-text product names and structured fields did not permit sufficiently reliable and harmonized classification across quarterly records. Absence of an extended-release token could not define immediate-release use.

The long-term analysis used the same spontaneous-reporting system and provides a temporal consistency assessment, not independent external replication. Future studies should use claims, electronic health records, or prospective cohorts with utilization denominators, reaction-specific onset dates, drug start dates, dose, formulation, renal function, symptom severity, competing etiologies, and patient-level follow-up. Such designs are required to estimate incidence, test causal susceptibility, and determine whether diarrhea-specific onset can inform monitoring.

## Conclusion

Metformin PS/SS reports showed a persistent but context-dependent strict-diarrhea reporting imbalance in contemporary and long-term AERS/FAERS data. The signal persisted under report-level sole-suspect restrictions, whereas active-comparator estimates were lower than the whole-database estimate. Measured differences between interval contributors and noncontributors reinforced the selected and non-reaction-specific nature of the interval findings. These results support reproducible signal characterization, not incidence, causal effect, severity, or a clinical monitoring window.

## Data Availability

Publicly available datasets were analyzed in this study. This data can be found here: FDA Adverse Event Reporting System (FAERS) quarterly data files, U.S. Food and Drug Administration. Direct link: https://www.fda.gov/drugs/fda-adverse-event-monitoring-system-aems/fda-adverse-event-monitoring-system-aems-latest-quarterly-data-files.

## Supplementary material

SUPPLEMENTARY FIGURE S1 Exploratory stratified reporting signals for metformin-associated strict diarrhea in contemporary FAERS. Points and confidence intervals summarize descriptive heterogeneity across strata defined by sex, age group, recorded serious-outcome-code status, reporter country, and occurrence country. Absence of a recorded serious-outcome code was not interpreted as confirmed non-seriousness. The reporter-country panel shows only estimable strata; the 37 reports with missing reporter country contained no metformin-exposed report and are documented in Supplementary Table S7. These comparisons do not identify causal susceptibility, event-specific severity, or clinically established high-risk groups. Abbreviation: FAERS, U.S. Food and Drug Administration Adverse Event Reporting System.

SUPPLEMENTARY FIGURE S2 Annual reporting odds ratios for metformin-associated outcomes in long-term AERS/FAERS data. Panel A shows strict diarrhea, the broad loose-stool phenotype, and the clean GI event composite. Panel B separately shows lactic acidosis-related metabolic safety events because their larger scale would compress the GI series. Both panels use logarithmic y-axes; 2026 includes Q1 only. Persistence supports temporal consistency, while variation is interpreted as reporting-system heterogeneity rather than clinical incidence. Abbreviations: AERS, Adverse Event Reporting System; FAERS, U.S. Food and Drug Administration Adverse Event Reporting System; GI, gastrointestinal; Q, quarter; ROR, reporting odds ratio.

SUPPLEMENTARY FIGURE S3 Empirical cumulative distribution of report-level event-date intervals among metformin PS/SS reports containing the strict-diarrhea preferred term and contributing valid exact dates. Curves show all 595 valid intervals, the report-level metformin PS-only subset of 390 intervals, and the sole-suspect subset of 400 intervals. The display is truncated at 365 days, but each curve retains its full group denominator; 75.1%, 75.9%, and 78.0% of the respective intervals were 365 days or shorter. The report-level PS-only definition required every metformin-matched drug record to be coded PS but did not exclude non-metformin suspect drugs. The sole-suspect definition excluded reports with a non-metformin PS/SS row. The event date is recorded at the report level and is not linked to individual reaction preferred terms. Therefore, these intervals are not diarrhea-specific onset times, Kaplan-Meier estimates, cumulative incidence, or a basis for a clinical monitoring window. Measured differences between interval contributors and noncontributors are reported in Supplementary Table S14. Abbreviations: d, days; n, number of reports; PS/SS, primary suspect or secondary suspect; PS, primary suspect.
